# A comparative study of the changes in the quality of life among patients with homonymous hemianopia, monocular blindness, or binocular diplopia

**DOI:** 10.1371/journal.pone.0329433

**Published:** 2025-08-26

**Authors:** Ji Woong Chang

**Affiliations:** 1 Department of Ophthalmology, Jeju National University Hospital, Jeju-si, Korea; 2 Graduate Program in Cognitive Science, Yonsei University, Seoul, Korea; Queen's University Belfast, UNITED KINGDOM OF GREAT BRITAIN AND NORTHERN IRELAND

## Abstract

To investigate changes in health-related quality of life (HRQOL), patients with homonymous hemianopia due to unilateral brain damage, monocular blindness with a best-corrected visual acuity of 20/1000 or less, or binocular diplopia for at least 6 months were enrolled. The 25-item National Eye Institute Visual Function Questionnaire (NEI VFQ-25) was used to evaluate the changes in HRQOL before and after the onset of the causative disease within and between the three study groups. The patients were asked to recall their state before disease onset and record it as accurately as possible for each question of the NEI VFQ-25, as well as score their current status. Among 43 included patients, 20 had homonymous hemianopia, 13 had monocular blindness, and 10 had binocular diplopia. The mean patient age was 56 years, and 12 patients were women (28%). A significant decrease in all HRQOL subscale scores was observed in the homonymous hemianopia and monocular blindness groups. In the binocular diplopia group, a lower number of subscales showed decreases in HRQOL. However, the changes in HRQOL before and after disease onset did not differ between groups. In conclusion, HRQOL change before and after homonymous hemianopia and monocular blindness did not differ. Considering the development background of the NEI VFQ-25, this questionnaire may not be sufficiently sensitive to distinguish HRQOL changes between the two disease groups. Hence, the NEI VFQ-25 might be useful for comparing HRQOL changes before and after the onset of a specific disease or before and after treatment, while not being sensitive to differentiate HRQOL differences between diseases. Thus, caution is needed when using the NEI VFQ-25 to compare HRQOL differences between disease groups.

## Introduction

The visual system is one of the most important sensory systems for assessing our environment and helps determine the response to stimuli or events. Therefore, abnormalities in the visual system can affect safety and independent social activities, eventually leading to a deterioration in the quality of life (QOL) [1,2]. The concept of health has gradually evolved to now include mental well-being, optimal social activities, and the ability to adequately perform functions, rather than to simply define health as the absence of physical abnormalities [3]. The World Health Organization defines health as “a state of complete physical, mental, and social well-being and not merely the absence of disease or infirmity” [4]; therefore, health is a broad spectrum of concepts consisting of the consequences of impairments, disabilities, and handicaps, as well as the individual’s perceived status of health and well-being [5]. Consequently, assessing the mental state and ability in addition to the function and performance of the body is necessary to evaluate the performance of social roles in an integrated manner. This is equally true in the field of ophthalmology [6]. A major challenge of assessing health-related QOL (HRQOL) is to develop measures for various HRQOL dimensions that meet rigorous standards of validity and reliability and reflect the practical constraints in clinical research settings. To measure HRQOL, a multidimensional approach is required, and four aspects are measured: physical, functional, psychological, and social [7]. Therefore, patient-reported outcomes (PROs) of the current state are important for evaluating the HRQOL of patients, and questionnaires are used to reflect PROs and HRQOL [8]. A method that can identify the extent of QOL changes after a specific eye disease has occurred is needed, but the changes in perceptions of visual functioning and HRQOL after the occurrence of an ophthalmic disease cannot be easily, accurately, and objectively measured [9].

Measuring visual acuity alone cannot effectively reflect the disabilities experienced by patients in daily life. For example, most patients with homonymous hemianopia have normal visual acuity although they experience considerable challenges when performing vision-dependent activities of everyday life. The best approach to obtain useful information regarding such problems is to acquire performance measures using real-life visual tasks such as reading, driving, depth perception, and mobility [1,10–12]. However, this is impractical in clinical settings because real-world situations are difficult to simulate and measure in clinical practice. Moreover, objective measurements of visual function alone cannot reflect the degree of visual impairment the patient experiences in daily activities [13]. By contrast, patients can be directly asked about self-perceived visual difficulties in their daily lives; however, these responses may not be objective and may be difficult to compare.

Thus, to measure HRQOL related to visual disabilities, not only objective values such as visual acuity or visual field measurements but also PROs and patient-reported symptoms are required [14]. Various questionnaires are used to evaluate subjective PROs and perceptions of patients. The validity of PROs, particularly whether they objectively evaluate what they are purported to measure, remains a limitation of questionnaires [15]. Therefore, selecting an appropriate questionnaire to measure patients’ HRQOL is of the essence.

Homonymous hemianopia, monocular blindness, and binocular diplopia are major visual system abnormalities that result in a decline in HRQOL, and studies have been conducted to determine the effect of each disability on HRQOL [16–21]. Although each of these visual impairments is recognized to negatively impact visual performance and psychosocial functioning, their comparative influence on HRQOL remains inadequately explored. Homonymous hemianopia restricts navigation and reading capabilities due to the loss of half of the visual field [10,19]. Monocular blindness results in the absence of stereopsis, leading to deficits in depth perception and an increased risk of accidents [22]. Diplopia interferes with both near and distance activities, including reading and driving, and is associated with significant psychological distress [16,17]. A comparative analysis of HRQOL among these diverse groups can provide valuable insight into the relative burden of disability and guide the formulation of targeted rehabilitation strategies. However, no prior study has compared the HRQOL among these conditions. Thus, this study aimed to measure and compare the changes in HRQOL after the occurrence of homonymous hemianopia, monocular blindness, and binocular diplopia.

## Materials and methods

This prospective study was approved by the Institutional Review Board and Ethics Committee of Inje University Ilsan Paik Hospital (approval number: 2020-08-007) and conducted between 08/10/2020 and 07/10/2023, according to the tenets of the Declaration of Helsinki. Written informed consent was obtained after explaining the purpose of the study.

The condition-specific eligibility criteria were as follows. 1) Homonymous hemianopia: Patients who had bilateral homonymous hemianopia with unilateral geniculocalcarine visual pathway damage confirmed using magnetic resonance imaging or computed tomography and whose homonymous hemianopia persisted for at least 6 months, as indicated by a 30–2 threshold with the Swedish Interactive Testing Algorithm Standard program on the Humphrey Visual Field Analyser (Carl Zeiss Meditec, Dublin, CA). The best-corrected visual acuity (BCVA) was 20/25 or better for both eyes. 2) Monocular blindness: Patients who had acquired monocular blindness with total visual field loss secondary to optic neuropathy or retinopathy with a BCVA of 20/1000 or worse in the blind eye and 20/25 or better in the healthy eye. 3) Binocular diplopia: Patients who had binocular diplopia for at least 6 months and because the symptoms are severe and unlikely to improve spontaneously therefore surgeries were scheduled to undergo surgery for diplopia. Patients who had other neurological disorders that may affect daily life, such as motor weakness and cognitive dysfunction, patients who could not understand the questionnaire, and patients with visual acuity below 20/25 in any eye except for the worse eye in those with monocular blindness were excluded.

Patients who visited the clinic for a scheduled examination and met the above criteria completed the 25-item National Eye Institute Visual Function Questionnaire (NEI VFQ-25) [23] at least 6 months after the onset of ophthalmic disability. The patients were interviewed using the Korean version of the NEI VFQ-25 [24]. To assess the extent of HRQOL changes associated with the development of homonymous hemianopia, monocular blindness, or diplopia, a modified version of the NEI VFQ-25 was administered during a single clinical visit. For each item, patients were asked to provide two ratings in parallel: one reflecting their perceived visual function before disease onset and the other reflecting their current condition. This parallel item-by-item design was intended to minimize recall bias and facilitate a direct within-subject comparison of pre- and post-disease status. Since these scales differed among the questions, the answers to each of the 25 questions were converted to a 0 − 100-point scale; 100 and 0 represented the best and worst possible scores, respectively [23]. The responses and questions were summarized by classifying them under 12 subscales as in the original NEI VFQ-25 [23]. To assess whether the time elapsed since disease onset influenced NEI VFQ-25 outcomes, patients were additionally categorized into three groups based on the interval between onset and questionnaire administration: 6–12 months, 12–24 months, and over 24 months. All medical records were completely anonymized, de-identified, and aggregated prior to data analyses.

### Statistical analysis

Statistical analyses were performed using the IBM Statistical Package for the Social Sciences software (version 25.0; IBM Corp., Armonk, NY, USA). The compositions of the groups were compared using Fisher’s exact test. The Mann–Whitney U test was used to compare the changes in NEI VFQ-25 scores before and after disease onset within each group and the score differences between two groups. The Kruskal–Wallis test was used to compare the changes in NEI VFQ-25 scores between three groups. Subscale score changes were compared across three time intervals within each diagnostic group using the Kruskal–Wallis test. Statistical significance was set at P < 0.05.

## Results

Forty-three patients, including 12 women (28%), were included in this study. The mean age of the study participants was 56 years. Homonymous hemianopia, monocular blindness, and binocular diplopia were observed in 20, 13, and 10 patients, respectively (Table 1). Sex, age, and the duration between the onset of conditions and administration of the questionnaire did not differ among the groups.

**Table 1:** Demographic characteristics of the participants.

	Homonymous hemianopia	Monocular blindness	Binocular diplopia	P
**N**	20	13	10	
**M:F**	14:6	12:1	5:5	0.087^a^
**Age (years)**	59.0 ± 20.1	48.0 ± 17.2	55.5 ± 19.1	0.266^a^
**DM (%)**	8 (40.0)	3 (23.1)	3 (30.0)	0.628^a^
**HTN (%)**	10 (50.0)	4 (30.8)	1 (10.0)	0.096^a^
**Causative disease**	Brain infarction: 12	Brain and orbital tumors: 3	CN4 palsy: 5	
	Brain hemorrhage: 4	NMO: 3	AACE: 4	
	AVM: 2	TON: 3	CN6 palsy: 1	
	Brain tumor: 1	NAION: 2		
	Brain abscess: 1	CRAO: 1		
		RD: 1		
**Time from disease onset to questionnaire administration (median [IQR], days)**	348 [180, 1287]	389 [180, 1338]	314.5 [251, 495]	0.989^b^

^a^Fisher’s exact test.

^b^Kruskal–Wallis test.

AACE, acute acquired comitant esotropia; AVM, arteriovenous malformation; CN4, 4^th^ cranial nerve; CN6, 6^th^ cranial nerve; CRAO, central retinal artery occlusion; DM, diabetes mellitus; F, female; HTN, hypertension; IQR, interquartile range; M, male; NAION, non-arteritic ischemic optic neuropathy; NMO, neuromyelitis optica; RD, retinal detachment; TON, traumatic optic neuropathy.

The differences between the subscale scores before and after disease onset in each group are shown in Table 2. In the homonymous hemianopia and monocular blindness groups, all subscale scores were significantly decreased. However, in the binocular diplopia group, most subscale scores showed a slight but significant decrease, except for color vision, ocular pain, and dependency. Moreover, the degree of changes in subscale scores in each group were compared to assess between-group differences. When comparing the HRQOL scores of the current status among groups, the scores after disease onset were not significantly different between pairs of groups with homonymous hemianopia, monocular blindness, or binocular diplopia (Table 3).

**Table 2:** Changes in health-related quality of life indexes before and after disease onset.

Subscale	Homonymous hemianopia		Monocular blindness		Binocular diplopia		Between groups
	Difference	P^a^	Difference	P^a^	Difference	P^a^	P^b^
**General health**	−32.0 ± 25.5	<0.001	−23.1 ± 18.0	0.005	−28.0 ± 23.5	0.021	0.577
**General vision**	−21.7 ± 15.4	<0.001	−34.6 ± 23.0	0.002	−16.7 ± 15.7	0.021	0.057
**Near vision**	–28.1 ± 20.8	0.001	−29.1 ± 21.5	0.004	−22.2 ± 20.3	0.021	0.967
**Distant vision**	−28.6 ± 18.8	<0.001	−27.8 ± 21.6	0.002	−18.9 ± 15.8	0.014	0.725
**Driving**	−45.7 ± 31.2	0.006	−37.0 ± 23.6	0.014	−33.7 ± 22.3	0.022	0.313
**Peripheral vision**	−27.5 ± 23.1	<0.001	−26.9 ± 18.7	0.003	−15.0 ± 16.6	0.034	0.199
**Color vision**	−23.3 ± 27.3	0.002	−16.7 ± 20.4	0.021	−8.3 ± 11.8	0.098	0.416
**Ocular pain**	−18.0 ± 17.9	0.001	−15.4 ± 12.0	0.005	−17.0 ± 20.6	0.054	0.663
**Role limitation**	−34.5 ± 28.7	0.001	−26.2 ± 26.3	0.009	−26.0 ± 24.6	0.022	0.175
**Dependency**	−25.7 ± 21.6	<0.001	−14.4 ± 17.6	0.014	−15.3 ± 22.2	0.098	0.498
**Social function**	−23.3 ± 20.0	<0.001	−23.7 ± 20.9	0.009	−13.3 ± 16.8	0.036	0.217
**Mental health**	−27.8 ± 21.4	<0.001	−20.4 ± 17.8	0.004	−17.5 ± 17.7	0.014	0.317

^a^Mann–Whitney U test for within-group comparisons.

^b^Kruskal–Wallis test for between-group comparisons.

**Table 3:** Differences in health-related quality of life indexes after disease onset between groups.

Subscale	Homonymous hemianopia − monocular blindness		Homonymous hemianopia − binocular diplopia		Monocular blindness − binocular diplopia		
	Difference (95% CI)	P^a^	Difference (95% CI)	P^a^	Difference (95% CI)	P^a^	P^b^
**General health**	−3.9 (−7.5, 15.4)	0.487	−3.0 (−11.3, 17.3)	0.661	1.0 (−16.3, 14.5)	0.901	0.266
**General vision**	8.4 (−20.7, 3.9)	0.169	−2.5 (−7.0, 12.0)	0.585	−11.0 (−2.6, 24.4)	0.107	0.871
**Near vision**	−3.7 (−11.2, 18.7)	0.611	−0.3 (−17.2, 17.8)	0.974	3.5 (−22.4, 15.5)	0.706	0.934
**Distant vision**	−1.0 (−13.5, 15.5)	0.890	1.7 (−15.1, 12.8)	0.800	2.7 (−17.8, 12.5)	0.720	0.872
**Driving**	−0.3 (19.7, 20.4)	0.973	−4.5 (−15.2, 24.1)	0.640	−4.1 (−15.7, 24.0)	0.664	0.398
**Peripheral vision**	0.83 (−14.9, 13.3)	0.905	−7.5 (−6.0, 21.0)	0.262	−8.3 (−5.6, 22.3)	0.228	0.098
**Color vision**	−1.9 (−15.9, 19.7)	0.826	−15.0 (−0.1, 30.1)	0.051	−13.1 (−3.2, 29.3)	0.109	0.372
**Ocular pain**	−8.0 (−3.2, 19.3)	0.156	−3.5 (−15.5, 22.5)	0.700	4.5 (−22.9, 14.1)	0.608	0.680
**Role limitation**	−7.2 (−10.4, 24.7)	0.410	−7.0 (−16.3, 30.3)	0.534	0.2 (−23.5, 23.2)	0.989	0.179
**Dependency**	−12.0 (−2.6, 26.6)	0.103	−13.7 (−5.1, 32.4)	0.142	−1.6 (−17.6, 20.9)	0.859	0.675
**Social function**	−2.7 (−12.7, 18.1)	0.722	−6.7 (−8.6, 21.9)	0.374	−4.0 (−12.7, 20.7)	0.626	0.433
**Mental health**	−6.5 (−7.4, 20.5)	0.345	−10.0 (−7.34, 27.3)	0.240	−3.5 (−14.6, 21.5)	0.691	0.266

^a^Mann–Whitney U test for between two groups comparisons.

^b^Kruskal–Wallis test for between three groups comparisons.

CI, confidence interval.

Effect sizes (Cohen’s d) were calculated to assess the magnitude of HRQOL decline within each group. Large effects were observed across all conditions: 1.54 for homonymous hemianopia, 1.71 for monocular blindness, and 1.10 for binocular diplopia, indicating substantial within-group reductions in NEI VFQ-25 total scores. A Kruskal–Wallis test on the total score differences showed no statistically significant difference among the three groups (H = 1.49, P = 0.470). Post-hoc power analyses confirmed that all groups had sufficient statistical power to detect the observed effects. To further explore the potential impact of time since disease onset on HRQOL outcomes, NEI VFQ-25 subscale score changes were compared across three time intervals (6–12 months, 12–24 months, and over 24 months) within each diagnostic group. No statistically significant differences were observed across these intervals for any of the subscales in homonymous hemianopia, monocular blindness, or binocular diplopia. The detailed results are presented in S1 Table.

## Discussion

Homonymous hemianopia, monocular blindness, and binocular diplopia are acquired, non-refractive visual impairments that share important features. All cause persistent disruption in spatial and functional vision, including difficulties with mobility, reading, driving, and daily activities. Each condition also imposes distinct psychosocial burdens. Moreover, these three disorders represent some of the most common and clinically significant causes of visual disability encountered in neuro-ophthalmology practice. These shared challenges and their clinical prevalence support the use of a unified patient-reported outcome measure like the NEI VFQ-25 to examine their impact on HRQOL. Therefore, this study investigated the changes in HRQOL among patients with homonymous hemianopia, monocular blindness, and binocular diplopia. The study results showed that patients with homonymous hemianopia and monocular blindness reported severe significant reductions in the scores for all subscales of the NEI VFQ-25. Although similar significant reductions were observed in most scores of the binocular diplopia group, the number of subscales showing reductions was lower than that in the homonymous hemianopia and monocular blindness groups. Homonymous hemianopia and monocular blindness are permanent, whereas diplopia effects can be alleviated by covering one eye, which may have accounted for the aforementioned difference. Additionally, all enrolled patients with diplopia were scheduled for surgery, which was expected to resolve the diplopia symptoms.

Several studies have investigated changes in QOL in patients with homonymous hemianopia [19,25], monocular blindness [21,26], and diplopia [27,28]. Chia et al. determined whether unilateral visual impairment had a measurable effect on QOL using the Short Form Health Survey (SF-36) in the Blue Mountains Eye Study, a cohort study conducted in Australia [21]. These authors concluded that unilateral visual impairment was associated with role limitation due to physical problems, social functioning, and role limitation due to emotional problems. The SF-36 was developed to evaluate multidimensional general health concepts and states in various diseases; however, it is not vision-specific [29]. In their study, unilateral visual impairment was defined as a BCVA of <6/12 in the worse eye and ≥6/12 in the better eye, which represents better visual conditions compared to the BCVA criteria used in the present study. Despite these differences, the results of Chia et al. also showed that vision problems such as unilateral visual impairment can affect psychophysical and mental health [21]. Hatt et al. compared differences in HRQOL for adults with strabismus between those with or without diplopia using open-ended questions derived from various studies. These authors found that patients with diplopia had problems predominantly related to negative feelings and everyday physical functioning [17]. Additionally, Wu-Chen et al. reported that the degree of diplopia was linearly correlated with worsening in the peripheral vision subscale of the NEI VFQ-25 [30].

Several studies have shown that visual field defects can reduce QOL [19,31,32]. Choi et al. compared the QOL of patients with homonymous hemianopia and monocular blindness and found a significant decrease in QOL for all subscales apart from general vision and ocular pain [25]. However, in the current study, the QOL of both groups did not differ in any of the subscales. This difference between the findings of the present study and those of Choi et al. may be attributed to differences in causative disease and survey timing. In the present study, patients with brain and orbital tumors, neuromyelitis optica, or central retinal artery occlusion were included in the monocular blindness group. These diseases can affect systemic conditions and increase mortality and morbidity rates, thus impacting QOL, which may have changed with the severity of the causative diseases. Moreover, as the average duration from disease onset to the survey was longer in the present study, the participants may have experienced a significant decrease in their QOL.

To my knowledge, no prior study has compared the QOL among patients with homonymous hemianopia, monocular blindness, and diplopia. Notably, the QOL scores of the groups before and after disease onset did not differ. These results may indicate that QOL measurements vary across studies depending on the characteristics of the participants included in each study.

Homonymous hemianopia and monocular blindness are not disease categories but a group of symptoms caused by various diseases. Therefore, HRQOL changes in patients with these disorders may vary depending on those causes. In the current study, patients with motor weakness or cognitive dysfunction that can seriously affect activities of daily living or those with other accompanying physical abnormalities were excluded. This implies that changes in HRQOL were mainly affected by ophthalmic abnormalities. Loss of stereopsis is the most important inconvenience of monocular blindness [33]; this inconvenience in daily life lowers the HRQOL [22,34]. Although prior reports demonstrated that monocular blindness at an early age results in normal binocular performance [35–37], the present study included only adults with monocular vision loss, who should have difficulties using monocular cues or adopting a method to compensate for the vision loss [34]. Thus, patients with monocular blindness reported significant decreases in HRQOL in the current study [38]. By contrast, patients with homonymous hemianopia are impaired in almost every aspect of their common activities of daily living, such as reading, driving, depth perception, and mobility [1,39,40]. Although only a few studies have investigated the relationship between stereopsis and homonymous hemianopia, it is reasonable to assume that stereopsis is poor in patients with homonymous hemianopia [41]. Since most areas of the binocular visual field are covered by a monocular visual field [42], patients with homonymous hemianopia are predicted to have greater difficulties in daily life than those with monocular blindness because they cannot recognize half of the visual field. Therefore, it was expected that the change in QOL would differ between the homonymous hemianopia and monocular blindness groups. However, the results of the present study did not confirm this assumption, and the reason for this was analyzed to be due to the characteristics of the questionnaire used in this study.

The NEI VFQ was initially developed to comprehensively measure and compare in clinical research vision-related, multidimensional HRQOL in patients with various eye diseases [2]. Subsequently, the NEI VFQ-25, a shorter form of the NEI VFQ, was developed to evaluate HRQOL more conveniently [23]. When originally developing the NEI VFQ questionnaire, the questions were designed by collecting and summarizing data on discomfort commonly experienced by patients with various ophthalmic diseases such as primary open-angle glaucoma, diabetic retinopathy, cataract, age-related macular degeneration, cytomegalovirus retinitis, and low vision from any cause. Given that the NEI VFQ-25 was not developed by clinicians or researchers, was not based on literature reviews, and was not intended to distinguish differences between disease groups, the questionnaire cannot identify outcome differences according to individual target diseases. Therefore, this questionnaire may not reflect subjective QOL changes depending on individual diseases. Therefore, it is thought that although the NEI VFQ-25 questionnaire evaluation in this study showed a measurable decline in QOL after homonymous hemianopia and monocular blindness, the degree of QOL decrease did not differ enough to distinguish between these two causes. These results are consistent with the limited discriminative capacity of the NEI VFQ-25. Although each condition affects visual function differently in clinical terms, the instrument did not detect significant differences in HRQOL change across groups. Post-hoc power analyses further indicated that the observed effect sizes were detectable with adequate statistical power across all groups, supporting the interpretation that the lack of between-group differences is more likely attributable to the limited sensitivity of the instrument than to insufficient statistical power. Among the subscales that showed significant within-group score reductions, General Health, Near Vision, Distance Vision, Driving, and Role Limitation tended to exhibit larger average declines. These domains appeared to contribute substantially to the overall decrease in HRQOL, as they consistently demonstrated marked reductions across all three patient groups.

This study has some limitations. First, the sample size was small. Second, the durations from disease onset to questionnaire administration varied although without reaching statistical significance. Therefore, the recall of the condition before disease onset may have been distorted in participants with longer duration. However, the QOL substantially decreased before and after disease onset on all subscales of the homonymous hemianopia and monocular blindness groups and on most subscales of the binocular diplopia group, and the difference in QOL scores at the time of questionnaire administration did not significantly differ between groups. This suggests that the time of questionnaire administration after disease onset did not significantly affect current QOL values and QOL changes. In the future, it will be necessary to conduct research to determine the differences in QOL immediately after disease onset and after a specific period following disease onset. Finally, the causative diseases in each group were highly diverse. Since the causative diseases did not only include eye-related conditions but also disorders that can have systemic effects, they may have resulted in QOL changes that cannot be accounted for by eye conditions alone.

## Conclusions

This study explored changes in HRQOL after the sequelae of various causative diseases such as homonymous hemianopia, monocular blindness, and binocular diplopia rather than examining the changes in HRQOL for specific eye diseases. Homonymous hemianopia, monocular blindness, and diplopia are symptoms of various underlying diseases, and the change in HRQOL was lesser after treatable diplopia than after permanent homonymous hemianopia or monocular blindness. However, neither the change in HRQOL between before and after disease onset nor the status after disease onset differed in the homonymous hemianopia and monocular blindness groups according to the NEI VFQ-25 results. Two interpretations of these results are possible. First, the difference in HRQOL between the homonymous hemianopia and monocular blindness groups may not be significant. Second, the questionnaire results may not be sensitive enough to distinguish HRQOL differences between various conditions. Considering the development background of the NEI VFQ-25 and the fact that in daily life, homonymous hemianopia is more difficult to adapt to or compensate for than monocular blindness, the NEI VFQ-25 may not be a suitable method for comparing disease groups. These findings suggest that the NEI VFQ-25 captures core functional burdens shared across anatomically distinct visual impairments, particularly in domains such as General Health, Near Vision, Distance Vision, Driving, and Role Limitation. However, given its limited sensitivity in detecting inter-group differences, the instrument may be more appropriate for evaluating within-group changes. Future research should focus on developing disease-specific assessment tools and on conducting longitudinal studies to better understand how patients adapt to various forms of visual loss over time. Considering these points, caution is needed when interpreting NEI VFQ-25 results.

## Supporting information

S1 Table NEI VFQ-25 subscale changes by time interval after disease onset. (DOCX)

S1 Data Data file. (XLSX)
