# Supplementary material for: A comparative study of the changes in the quality of life among patients with homonymous hemianopia, monocular blindness, or binocular diplopia
Source: PLoS One. 2025 Aug 26;20(8):e0329433. doi: 10.1371/journal.pone.0329433 (PMC12380287; doi:10.1371/journal.pone.0329433)
Supplement: S1 Table — (DOCX) [file pone.0329433.s001.docx]

**S1 Table. NEI VFQ-25 subscale changes by time interval after disease onset.**

| **Patient group** | **Subscale** | **Score changes** | | |  |
| --- | --- | --- | --- | --- | --- |
|  |  | 6–12 months | 12–24 months | >24 months | P^a^ |
| **Homonymous hemianopia** | General health | -25.0 ± 17.7 | -32.1 ± 12.4 | -31.8 ± 14.0 | 0.910 |
|  | General vision | -30.0 ± 14.1 | -35.5 ± 15.2 | -33.7 ± 13.9 | 0.850 |
|  | Near vision | -35.0 ± 13.5 | -38.2 ± 14.7 | -36.9 ± 12.9 | 0.830 |
|  | Distant vision | -32.5 ± 15.0 | -36.7 ± 15.3 | -35.2 ± 13.6 | 0.920 |
|  | Driving | -40.0 ± 17.3 | -41.2 ± 16.6 | -39.8 ± 15.2 | 0.890 |
|  | Peripheral vision | -33.0 ± 13.2 | -36.2 ± 13.5 | -35.1 ± 14.3 | 0.950 |
|  | Color vision | -5.0 ± 4.1 | -7.1 ± 7.2 | -6.6 ± 5.9 | 0.930 |
|  | Ocular pain | -10.0 ± 7.1 | -14.3 ± 10.2 | -13.6 ± 9.7 | 0.890 |
|  | Role limitation | -31.0 ± 14.7 | -34.1 ± 14.2 | -33.2 ± 13.1 | 0.910 |
|  | Dependency | -12.5 ± 8.8 | -15.7 ± 9.7 | -14.8 ± 8.9 | 0.970 |
|  | Social function | -28.0 ± 12.0 | -31.3 ± 13.8 | -30.7 ± 14.5 | 0.860 |
|  | Mental health | -29.0 ± 13.2 | -32.9 ± 13.7 | -31.5 ± 15.0 | 0.900 |
| **Monocular blindness** | General health | -27.5 ± 15.0 | -30.3 ± 13.2 | -29.1 ± 12.7 | 0.930 |
|  | General vision | -28.0 ± 14.5 | -31.2 ± 12.9 | -30.7 ± 13.4 | 0.910 |
|  | Near vision | -29.0 ± 13.7 | -31.4 ± 14.1 | -30.8 ± 14.3 | 0.920 |
|  | Distant vision | -27.5 ± 13.9 | -30.6 ± 14.2 | -30.2 ± 14.5 | 0.900 |
|  | Driving | -32.0 ± 15.1 | -34.6 ± 15.4 | -34.0 ± 15.6 | 0.940 |
|  | Peripheral vision | -28.0 ± 13.4 | -30.8 ± 13.8 | -30.3 ± 14.1 | 0.920 |
|  | Color vision | -4.0 ± 4.0 | -4.4 ± 4.5 | -4.2 ± 4.7 | 0.910 |
|  | Ocular pain | -9.0 ± 8.2 | -10.5 ± 9.1 | -10.1 ± 9.5 | 0.920 |
|  | Role limitation | -27.0 ± 14.6 | -29.8 ± 14.9 | -29.4 ± 15.1 | 0.900 |
|  | Dependency | -10.5 ± 7.9 | -11.7 ± 8.3 | -11.4 ± 8.5 | 0.910 |
|  | Social function | -25.0 ± 12.1 | -27.8 ± 12.5 | -27.3 ± 13.0 | 0.930 |
|  | Mental health | -26.0 ± 13.3 | -28.7 ± 13.8 | -28.2 ± 14.2 | 0.920 |
| **Binocular diplopia** | General health | -18.8 ± 12.3 | -20.2 ± 10.1 | -19.6 ± 11.2 | 0.980 |
|  | General vision | -20.5 ± 13.2 | -21.7 ± 12.5 | -22.1 ± 12.8 | 0.970 |
|  | Near vision | -22.0 ± 12.5 | -23.8 ± 13.1 | -22.9 ± 14.0 | 0.960 |
|  | Distant vision | -21.5 ± 13.0 | -23.3 ± 13.6 | -22.7 ± 13.9 | 0.950 |
|  | Driving | -24.0 ± 14.0 | -25.2 ± 14.3 | -24.7 ± 14.6 | 0.960 |
|  | Peripheral vision | -21.0 ± 12.2 | -22.6 ± 12.8 | -22.0 ± 13.2 | 0.980 |
|  | Color vision | -3.0 ± 3.2 | -3.6 ± 3.7 | -3.4 ± 3.9 | 0.970 |
|  | Ocular pain | -8.0 ± 6.1 | -9.2 ± 7.3 | -7.8 ± 8.0 | 0.960 |
|  | Role limitation | -21.0 ± 13.0 | -22.8 ± 13.4 | -22.2 ± 13.7 | 0.970 |
|  | Dependency | -7.5 ± 6.3 | -8.4 ± 7.1 | -8.1 ± 7.5 | 0.990 |
|  | Social function | -19.0 ± 11.7 | -20.7 ± 12.2 | -20.1 ± 12.7 | 0.960 |
|  | Mental health | -20.0 ± 12.5 | -21.9 ± 12.9 | -21.3 ± 13.1 | 0.970 |

^a^Kruskal–Wallis test for between three periods comparisons.

M, months
